# Efficient Ultrasound-Assisted Extraction of Bioactive Molecules from Brown Macroalga *Sargassum horneri*: Optimal Extraction, Antioxidant and Cytotoxicity Evaluation

**DOI:** 10.3390/ijms26062749

**Published:** 2025-03-19

**Authors:** Yunseok Song, Jeongho Lee, Hyeok Ki Kwon, Minji Kim, Soeun Shin, Seunghee Kim, Hyerim Son, Chulhwan Park, Hah Young Yoo

**Affiliations:** 1 Department of Biotechnology, Sangmyung University, Seoul 03016, Republic of Korea; 2 Department of Chemical Engineering, Kwangwoon University, Seoul 01897, Republic of Korea

**Keywords:** *Sargassum horneri*, ultrasound-assisted extraction, optimization, antioxidant, tannin, phenol

## Abstract

*Sargassum horneri* (SH) is a promising marine bioresource for producing bioactive compounds. Recently, the biological functions (including anti-inflammatory, antioxidant, and anticancer activities) of SH extracts have been revealed; however, efficient extraction processes to produce bioactive molecules (such as tannin and phenol) have not been carefully designed. In this study, the ultrasound-assisted extraction process was optimized based on the response surface methodology (RSM) to efficiently produce tannin and phenol from SH. Significant RSM models (*p* < 0.05) for predicting tannin and phenol yields were developed, and prethanol A concentration, temperature, and solid loading were significantly affected by tannin or phenol yield (*p* < 0.05). Following numerical optimization, the tannin and phenol yields achieved 14.59 and 13.83 mg/g biomass, respectively, under optimal conditions (39.1% solvent, 61.9 °C, 52.0 g/L solid loading, and 49.0% amplitude), similar to the model-predicted values (12.95 and 13.37 mg/g, respectively). Then, time profiling under optimal conditions determined the optimal time as 10.0 min, resulting in the highest yield (15.88 mg tannin and 14.55 mg phenol/g). The extracts showed antioxidant activity (IC_50_: 79.86 μg/mL) comparable to that of ascorbic acid (vitamin C). It was found to be particularly non-toxic, raising its potential as a functional ingredient in food or cosmetics.

## 1. Introduction

Brown macroalgae are attracting attention as a promising source of bioactive molecules such as phenolics, polysaccharides, and vitamins [1,2,3]. In particular, tannins and phenols have been reported as natural molecules of brown macroalgae, with various biological activities such as antioxidant, anticancer, antidiabetic, and antiviral activities [4]. Bioactive substances extracted from brown macroalgae have been applied to develop various products such as food and cosmetics, thereby promoting the discovery of next-generation bioactive molecules [5,6,7]. In recent years, research has been actively conducted on feedstock exploration, extraction process design, formulation, and application [1,8,9].

Since 2015, *Sargassum horneri* (SH) has been entering Jeju Island and the coast of the Korean Peninsula between February and May each year, causing significant damage to aqua-farming sites and navigation [10]. In particular, it causes damage to aquaculture facilities, reduces the quality of farmed organisms, and creates odors due to spoilage, all of which are costly [11]. While some of the large amounts of SH are used as agricultural fertilizer or livestock feed, most of them are landfilled or incinerated, which requires diversified sustainable use [12]. The following applications were reported for the sustainable use of SH: bioactive ingredients [13], biodegradable packaging films [14], and a potential therapeutic drug for treating particulate matter-exacerbated allergic asthma [15].

SH extracts contain various bioactive compounds such as phenol, tannin, fucoxanthin, and polysaccharides, and various bioactivities of SH extracts (including anti-inflammatory, antioxidant, and anticancer activities) have been identified [16,17,18,19,20]. Although optimization of the extraction process for some bioactive compounds (fucoxanthin and polysaccharide) has been conducted [20,21], the process design and optimization for efficient tannin and phenol production from SH have not been carefully performed. Therefore, it is necessary to design a process for efficiently extracting bioactive molecules from SH.

Bioactive molecules including phenols and tannins can be recovered from biomass by conventional methods including maceration and decoction [22,23]. Maceration is a simple method that is easy to scale up, but it must be operated for a long time to obtain a high extraction yield [24,25,26]. Since the decoction requires thermal interference, it is only suitable for extracting thermally stable compounds. [25]. Accordingly, non-conventional extraction methods such as ultrasound-assisted extraction have been developed to address high energy consumption and long extraction time [27]. By applying ultrasound-assisted extraction, bioactive compounds can be efficiently extracted from biomass under mild conditions because of the cavitation phenomenon [28,29]. In addition, recent studies successfully performed the scale-up of the ultrasound-assisted extraction process after optimization at the laboratory scale, with satisfactory extraction yields [30,31].

This study aimed to design and optimize an ultrasound-assisted extraction process to efficiently recover bioactive molecules such as tannins and phenols from SH. In the extraction process, considering applications such as food and cosmetics, prethanol A (ethanol) was utilized as a solvent. Major process parameters such as solvent concentration, extraction temperature, solid loading, and amplitude were selected and investigated for response surface modeling and optimization. Furthermore, antioxidant activity and cytotoxicity properties were investigated to evaluate the practical applications of SH.

## 2. Results and Discussion

### 2.1. Development of Regression Models for Predicting Tannin and Phenol Yields from Sargassum horneri

The objective was to optimize ultrasound-assisted extraction process parameters in order to maximize the extraction yield of tannins and phenols from SH. To optimize extraction process parameters, a model must be developed that can predict tannin and phenol extraction yields within designed extraction conditions. A regression model was developed to predict the extraction yield of tannin and phenol through extraction experiments designed by Design Expert (Table 1). Among the results of the extraction experiments, the highest tannin extraction yield (12.63 mg/g biomass) was observed under Std #20 (40% solvent, 70 °C, 100 g/L, and 60% amplitude). These results suggest that high temperatures are required for efficient tannin recovery from SH, but high concentrations of the solvent are not essential. The highest extraction yield of phenol (13.16 mg/g biomass) was observed under Std #4 (60% solvent, 60 °C, 75 g/L, and 40% amplitude). These results suggest that there is a partial difference, especially solvent concentration, between tannin and phenol recovery, requiring multi-objective optimization to produce bioactive extracts from SH.

Based on the results in Table 2, a regression analysis was performed to predict tannin and phenol extraction yields, and the following Equations (1) and (2) were derived, respectively.

Tannin yield (mg/g biomass) = 11.11 − 0.6707A + 0.7481B − 0.6783C − 0.0221D + 0.0344AB + 0.0472AC − 0.1515AD − 0.1927BC − 0.1669BD − 0.0165CD − 1.1A^2^ − 0.1944B^2^ − 0.2867C^2^ + 0.0591D^2^ (1)

Phenol yield (mg/g biomass) = 8.22 + 0.2611A + 0.6596B − 1.52C + 0.435D + 0.9136AB − 0.5149AC + 0.47AD − 0.9713BC − 0.3394BD − 0.1543CD − 0.5895A^2^ + 0.0149B^2^ − 0.0746C^2^ − 0.0805D^2^ (2)

An analysis of variance (ANOVA) was performed to assess the statistical significance of regression model Equations (1) and (2) (Table 2 and Table 3). In general, *p*-values less than 0.05 indicate that the prediction model and model terms are statistically significant. In the case of Equation (1), solvent conc. (A, *p*-value = 0.001), extraction temp. (B, *p*-value = 0.0004), and solid loading (C, *p*-value = 0.0009) were significant parameters for tannin yield. On the contrary, it was confirmed that amplitude (D, *p*-value = 0.8949) and all interaction effects did not have a significant effect (*p*-value > 0.05) on tannin yield. Among the square terms, an indicator that evaluates the effect on tannin extraction yield when the value of each parameter increases to the limit, only A^2^ (*p*-value < 0.0001) was found to have a statistically significant effect. In the case of Equation (2), the single parameters extraction temp. (*p*-value = 0.0399) and solid loading (*p*-value = 0.0001), and the interaction effects AB (*p*-value = 0.0225) and BC (*p*-value = 0.0163) were confirmed as significant parameters for phenol yield. Single parameter A was confirmed to be not statistically significant, but the square term A^2^ (*p*-value = 0.0483) was found to have a significant impact, requiring reflection of parameter A into the model equation for numerical optimization [4].

In the results of the ANOVA, the R^2^ value is an indicator of the degree of agreement between predicted and observed values [32]. As the R^2^ value approaches 1, it can be judged that the prediction accuracy of the regression model is high. However, as the number of independent parameters increases, the R^2^ value tends to increase regardless of the prediction accuracy. To prevent such overfitting errors, the adjusted R^2^ value can be used as an indicator and was found to be 0.7692 for the tannin yield prediction model and 0.6040 for the phenol yield prediction model. To improve the prediction accuracy of the model, a model reduction was performed to remove model terms that did not have a statistically significant impact. As a result, the following reduced model Equations (3) and (4) were derived.

Tannin yield (mg/g biomass) = 10.73 − 0.6707A + 0.7481B − 0.6783C − 1.05A^2^ (3)

Phenol yield (mg/g biomass) = 7.63 + 0.2611A + 0.6596B − 1.52C + 0.9136AB − 0.9713BC(4)

The results of the ANOVA on the improved model Equations (3) and (4) are summarized in Table 4 and Table 5. By removing statistically insignificant parameters (D, AB, AC, AD, BC, BD, CD, B^2^, C^2^, and D^2^) from the initial model (1), the reduced model (3) was derived, and this model was selected as the final model to predict the tannin yield. An improved model (4) without the insignificant parameters (D, AC, AD, BD, CD, A^2^, B^2^, C^2^, and D^2^) of the initial model (2) was selected as the final model to predict the phenol yield.

### 2.2. Effects of Extraction Parameters on Tannin and Phenol Extraction Yields from Sargassum horneri

**Figure 1:**
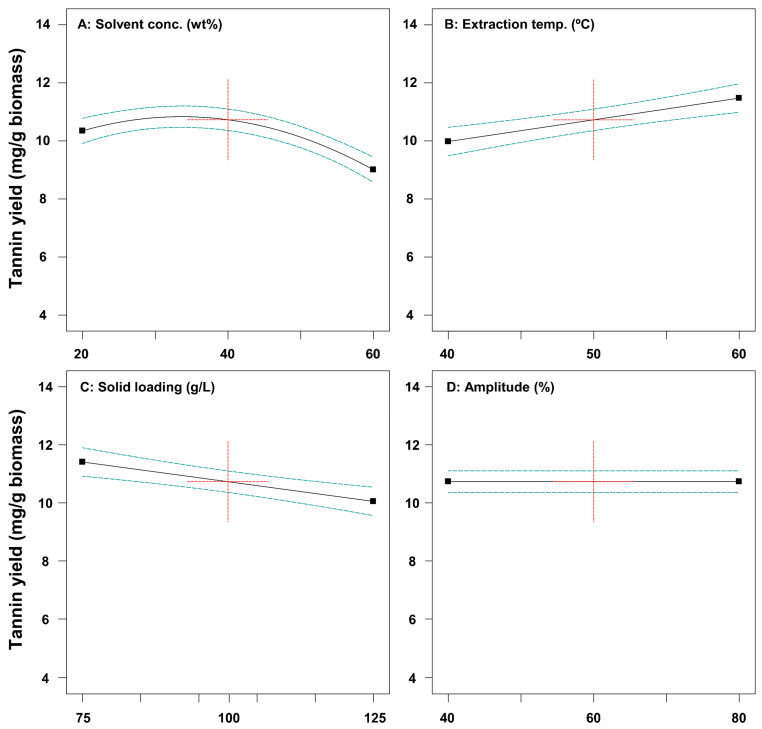
Plots representing the effects of solvent concentration (**A**), extraction temperature (**B**), solid loading (**C**), and amplitude (**D**) on tannin yield from *Sargassum horneri*.

**Figure 2:**
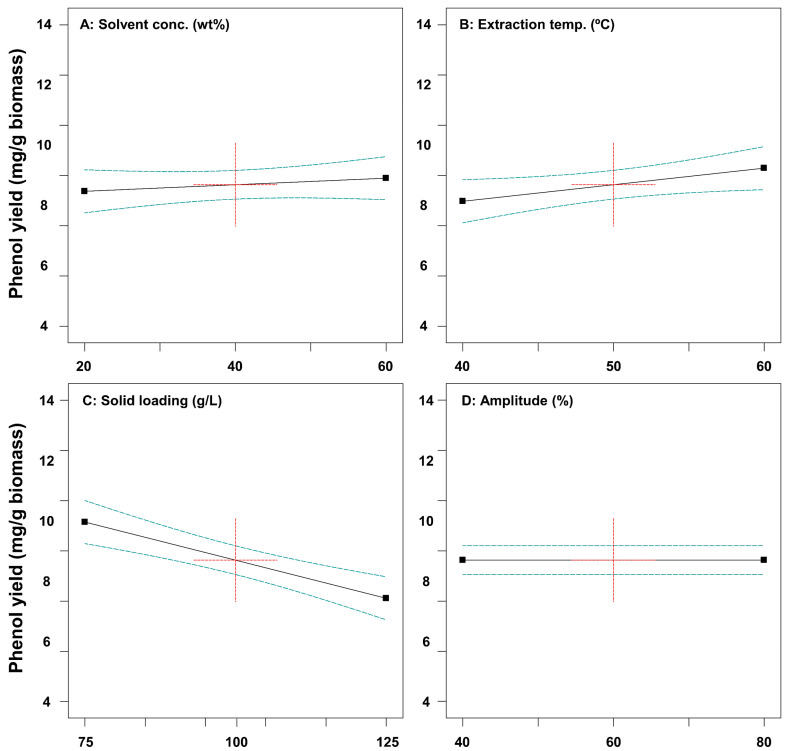
Plots representing the effects of solvent concentration (**A**), extraction temperature (**B**), solid loading (**C**), and amplitude (**D**) on phenol yield from *Sargassum horneri*.

The effects of ultrasound-assisted extraction process parameters on the extraction yield of tannin and phenol from SH were investigated. Figure 1 shows the one-factor effects on tannin extraction yield. A solvent concentration higher than about 40% decreased tannin yield from SH. Gam et al. [33] reported that as the solvent concentration increases, the total phenol content tends to increase and then rapidly decrease. The higher temperature resulted in a higher tannin and phenol yield from SH (Figure 1 and Figure 2). Kong et al. [34] showed that a high temperature leads to a higher yield extraction of phenolic compounds than a low temperature. The extraction process for high-solid loading resulted in decreased tannin and phenol yield (Figure 1 and Figure 2). In ultrasound-assisted extraction processes with low solid loading, the interaction between solids and liquids is more active, and the diffusion of the bioactive compounds from solid to liquid is more efficient [35].

### 2.3. Optimization of Extraction Parameters for Efficient Tannin and Phenol Recovery from Sargassum horneri

The developed models (3) and (4) can predict response values (i.e., tannin yield and phenol yield) within the designed range; thus, these were used in the numerical optimization. The targets were both maximizations of tannin yield and phenol yield from SH, and the results are shown in Table 6. The optimal extraction conditions were derived as follows: solvent concentration, 39.1%; extraction temperature, 61.9 °C; solid loading, 52.0 g/L; and amplitude, 49.0%. Under these conditions, the tannin and phenol yields were predicted to be 12.95 and 13.37 mg/g biomass, respectively. To verify the prediction accuracy of the model, a verification experiment was conducted. As a result, the extraction yield of tannin and phenol was determined to be about 14.59 and 13.83 mg/g biomass, similar to the model-predicted values (*p*-value > 0.05). Therefore, the prediction model is suitable for predicting tannin and phenol yields. This study is significant in that an optimization model was developed for the first time.

**Figure 3:**
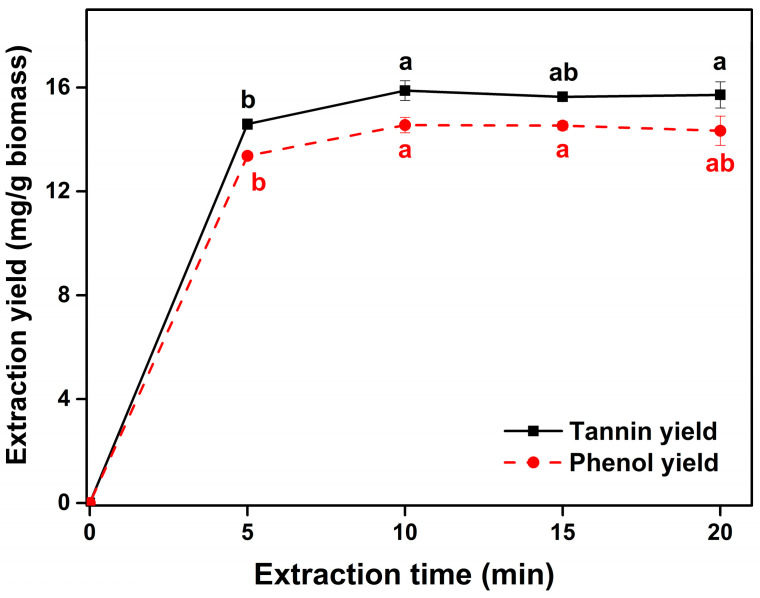
The time profile of tannin and phenol extraction yield from *Sargassum horneri* under optimal extraction conditions (39.1% solvent concentration, 61.9 °C, 52 g/L solid loading, and 49% amplitude). Data with different letters are significantly different (*p* < 0.05).

To further increase the tannin and phenol yields from SH, it is necessary to optimize the extraction time; thus, extraction profiling was performed for 20.0 min under the RSM-determined optimal conditions (Figure 3). As a result, the extraction yields of tannin and phenol showed an increasing trend up to 10.0 min but did not significantly increase thereafter (*p* < 0.05). Therefore, the optimal extraction time was determined to be 10.0 min. The extraction yield was determined to be about 15.88 ± 0.38 mg tannin and 14.55 ± 0.30 mg phenol per g biomass under the final conditions: solvent concentration, 39.1%; extraction temperature, 61.9 °C; solid loading, 52.0 g/L; amplitude, 49.0%; and extraction time, 10.0 min. Overall, this study designed an optimal ultrasound-assisted extraction process for producing bioactive compounds from SH with a high yield. The produced tannin and phenol contained in the SH extracts at 42.15 and 39.95 mg/g, respectively. Kim et al. [17] produced SH extracts by immersing 10 g of dried SH in 500 mL of ethanol (70%) while stirring at room temperature for 12 h, and the total phenol content of the extracts was 8.52 ± 2.64 mg GAE/g. Therefore, the ultrasound-assisted extraction process is efficient for preparing phenol-rich SH extracts.

### 2.4. Antioxidant of Sargassum horneri Extracts

ABTS radical scavenging activity was determined to evaluate the antioxidant activity of the SH extracts (Table 7). The results of the radical scavenging activity (%) of the extracts were represented as IC_50_ values (μg/mL). The ABTS IC_50_ value of the SH extracts was determined to be about 79.86 μg/mL, which was about 68% activity compared to that of ascorbic acid (ABTS IC_50_: 54.37 μg/mL). This antioxidant activity may be attributed to compounds such as tannins and phenols. Dang et al. [36] evaluated the ABTS radical scavenging activities of *Sargassum* extracts such as *S. vestitum*, *S. linearifolium*, and *S. podocanthum* extracts (at 0.06 mg/mL), and the activities were determined to be 31.71, 2.02, and 13.30 mg Trolox equivalent (TE)/g extract, respectively. These were 30.41%, 1.94%, and 12.76% activity compared to that of ascorbic acid (104.27 mg TE/g at 0.06 mg/mL) [36]. In the case of *S. coriifolium* extracts, the ABTS IC_50_ value was determined to be 2.19 mg/mL, which was 7.31% activity of that of ascorbic acid (0.16 mg/mL) [37]. Meanwhile, in addition to tannins and phenols, compounds that contribute to antioxidant activity may exist in SH extracts [38,39], requiring the further identification of bioactive molecules before practical applications. This study confirmed that the SH extracts are expected to have a high potential as a natural antioxidant among various *Sargassum* extracts. The produced SH extracts with antioxidant activity can be applied to the development of antioxidant foods and antioxidant cosmetics. For instance, Park et al. [40] developed a functional ingredient containing SH extracts for food applications, with antioxidant and anti-obesity effects. Choung et al. [41] used SH extracts to develop a cosmetic with antioxidant and skin-whitening effects.

### 2.5. Effect of Sargassum horneri Extracts on the L929 Cell Viability

**Figure 4:**
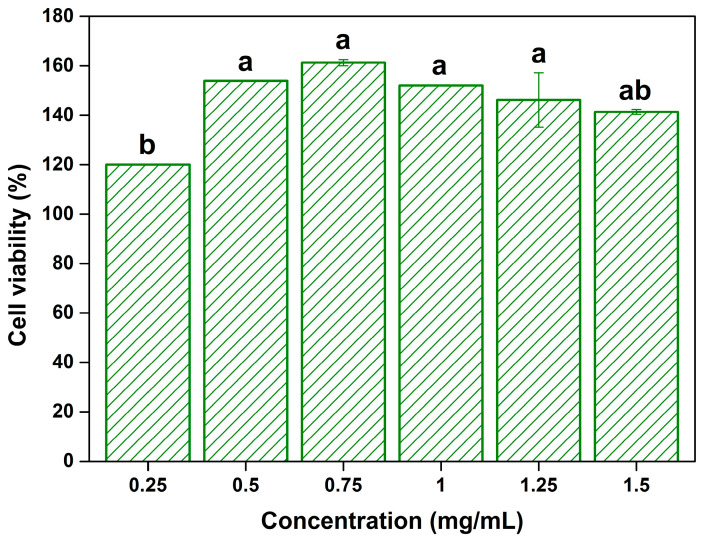
The effect of *Sargassum horneri* extracts on the L929 cell viability. Data with different letters are significantly different (*p* < 0.05).

Figure 4 shows the cell viability of the SH extract-treated normal L929 fibroblast cells. Cell viability increased at all tested concentrations (0.25–1.5 mg/mL), with a maximum of 161.3% (at 0.75 mg/mL). In particular, cell viability in the extracts treatment group at concentrations of 0.5–1.25 mg/mL was significantly increased compared to that in the 0.25 mg/mL group (*p* < 0.05). The improved cell viability due to the addition of SH extracts can be related to the antioxidant activity of SH extracts. According to Li et al. [42], proteins secreted by the L929 cell lines are responsible for reactive oxygen species (ROS) production, and increased ROS levels have been reported to decrease cell viability. Turan et al. [43] evaluated the cytotoxicity of curcumin-loaded nano-fibrous membranes, showing an increased L929 cell viability (at 24 h measurement), and they presumed that this increase could be related to the ability of curcumin to prevent the formation of ROS. Therefore, the increased cell viability in this study can be attributed to the antioxidant activity of the SH extracts, but the related mechanisms should be investigated in detail, considering the presence of other substances besides antioxidants such as tannins and phenols. The present study highlights the non-toxicity of the SH extracts, which indicates their potential safety for applications.

## 3. Materials and Methods

### 3.1. Materials

SH was purchased from Parajeju (Jeju, Republic of Korea), washed with fresh water, naturally dried for two days, and ground. Na_2_CO_3_ (CAS No. 144-55-8) was purchased from DaeJung Chemicals & Metals (Siheung, Republic of Korea). Prethanol A (CAS No. 64–17-5) and tannin acid (CAS No. 1401-55-4) were purchased from Duksan Chemical (Ansan, Republic of Korea). Folin-Ciocalteu reagent, NaNO_2_ (CAS No. 7632-00-0), K_2_S_2_O_8_ (CAS No. 7727-21-1), gallic acid (CAS No. 149-91-7), ascorbic acid (CAS No. 50-81-7), 2,2’-Azino-bis (3-ethyl-benzothiozoline)-6-sulfonic acid (ABTS) (CAS NO. 28752-68-3), and potassium persulfate (CAS No. 7727-21-1) were purchased from Sigma-Aldrich (St. Louis, MO, USA).

### 3.2. Experimental Design and Statistical Optimization for Maximizing Tannin and Phenol Extraction Yields

RSM modeling and optimization were performed to maximize tannin and phenol yield from SH. Ultrasound-assisted extraction was performed by an Ultrasonic processor VCX–130 (Sonics and Materials Inc., Newton, CT, USA) with a 6 mm diameter probe (amplitude: 120 μm). The temperature was controlled using a water bath during the extraction process to prevent undesired thermal effects. For the process optimization, prediction model development, statistical model evaluation, and numerical optimization were performed based on the response surface methodology and central composite design using Design-Expert software version 7 (Stat-Ease Inc., Minneapolis, MN, USA) [26,44]. The following Table 8 lists the extraction parameters selected as a major factor of the process.

The designed 30 conditions for RSM modeling are listed in Table 1. The volume of extraction solvent was fixed at 10 mL, and extraction was performed in a 50 mL plastic tube. All extractions were performed for 5.0 min. After the extraction, the concentrations of tannin and phenol in the extracts were quantified. These values were represented as tannin and phenol yields (mg/g biomass). The prediction models for tannin yield and phenol yield were developed using the following Equation (5). The meaning of each symbol in Equation (5) is as follows: *Y* is the response value (tannin yield or phenol yield); *X*_i_ and *X_j_* are independent parameters; *β*_0_ is the intercept; *β_i_*, *β_ij_* and *β_ii_* are regression coefficients; ϵ is the error term; and *k* is the number of independent parameters [45]. The statistical significance of the developed prediction model was tested by ANOVA.

(5) $$Y=\beta_{0}+\sum_{i=1}^{k}\beta_{i}X_{i}+\sum_{i=1}^{k-1}\sum_{i=i+1}^{k}\beta_{ij}X_{i}X_{j}+\sum_{i=1}^{k}\beta_{ii}X_{i}^{2}+\epsilon$$

### 3.3. Measurement of Tannin Concentration

The tannin concentration in the extracts was quantified by the Folin–Ciocalteu colorimetric Method. For tannin quantification, 20 μL of the extracts, 900 μL of distilled water, and 25 μL of the Folin–Ciocalteu reagent were mixed. After vertexing, 50 μL of the Na_2_CO_3_ solution (20%) was added and reacted at 24 °C for 30.0 min. After the reaction, the optical density (OD) of the mixture was measured at 700 nm. The results are expressed as mg tannic acid equivalent per g biomass.

### 3.4. Measurement of Phenol Concentration

The phenol concentration in the extracts was quantified by the Folin–Ciocalteu colorimetric method. For phenol quantification, 10 μL of the extracts, 790 μL of distilled water, and 50 μL of the Folin–Ciocalteu reagent were mixed and reacted at 30 °C for 8.0 min. After the reaction, 150 μL of 20% Na_2_CO_3_ (20%) was added and reacted at 25 °C for 1 h. The OD at 765 nm was measured [46]. The results are expressed as mg gallic acid equivalent per g biomass.

### 3.5. Evaluation of ABTS Cation Radical Scavenging Activity

The ABTS cation radical scavenging activity of the SH extracts was evaluated. The ABTS^•+^ solution was prepared by mixing the ABTS solution and 2.45 mM potassium persulfate in a 1:1 ratio and then diluted with methanol to prepare the solution with 1.0 of absorbance at 734 nm. An amount of 50 μL of the sample was added to 950 μL of the ABTS^•+^ solution, and the mixture was incubated at 25 °C for 30.0 min. The OD_734 nm_ was measured, and the ABTS radical scavenging activity (%) was calculated using the following Equation (6). The results were expressed as IC_50_ (μg/mL), which is the concentration required to scavenge 50% of the ABTS cation radicals.

(6) $$\mathrm{ABTS}\;\mathrm{cation}\;\mathrm{radical}\;\mathrm{scavenging}\;\mathrm{activity}\;(\%)=1-\frac{OD734\;nm\;of\;sample}{OD734\;nm\;of\;control}$$

### 3.6. Culturing of L929 Fibroblast Cells

The mouse lung fibroblast cell L929 was procured for European Collection of Cell Cultures (ECACC 85011425). The cells were cultured with Dulbecco’s modified Eagle medium (DMEM) containing 10% (*v*/*v*) fetal bovine serum, 100 U/mL of penicillin, 100 μg/mL of streptomycin, and 250 ng/mL of amphotericin B in a 5% CO_2_ incubator at 37 °C.

### 3.7. WST-1 Assay for Cytotoxicity Evaluation

The effect of the extracts on cell viability was determined by a WST-1 assay using a Viability Assay Kit (CELLO MAX TM, Anyang-si, Gyeonggi-do, Republic of Korea). For the assay, 100 μL of DMEM containing 1 × 10^4^ cells/well was dispensed into a 96-well plate and incubated in a 5% CO_2_ incubator for 24 h. After incubation, 1% of deionized water (as a control group) or the extracts diluted with deionized water was added to each well at 1% of the medium volume to reach 0.25–1.5 mg/mL and incubated in a 5% CO_2_ incubator for 48 h. Then, 10 μL of the WST-1 reagent was added to each well and incubated for 4 h, and the absorbance (*A*) was measured at 450 nm using a microplate reader (BIO-TEK, Los Angeles, CA, USA). Cell viability was determined using Equation (7).

Cell viability (%) = *A*_1_**/***A*_0_ × 100 (7)

where *A*_0_ and *A*_1_ are the absorbance of the control and experimental groups, respectively.

### 3.8. Statistical Analysis

All experiments were performed in triplicate, and all data are presented as mean ± standard deviation. The data related to the time profiling experiment and cytotoxicity evaluation were subjected to an analysis of variance (ANOVA) using SPSS Statistics 27 software (IBM-SPSS Inc., Chicago, IL, USA). The significant difference between each group was tested using Tukey’s test at 95% significance.

## 4. Conclusions

In this study, highly antioxidant and non-toxic bioactive extracts were successfully produced from SH in a high yield through response surface modeling and optimization of the ultrasound-assisted extraction process. In the RSM study, prethanol A concentration, extraction temperature, and solid loading were determined to be parameters that had a statistically significant effect (*p* < 0.05) on tannin yield or phenol yield. The RSM model-based optimization and time profiling studies maximized tannin yield and phenol yield from SH (15.88 mg and 14.55 mg per g biomass, respectively). The SH extracts, rich in tannins and phenols, showed a high antioxidant capacity (IC_50_: 79.86 μg/mL, 68% compared to ascorbic acid), demonstrating its high potential as a natural antioxidant. Moreover, the SH extracts were non-toxic to normal L929 fibroblast cells up to high concentrations (1.5 mg/mL), making it a suitable material for applications such as food and cosmetics. The ultrasound-assisted extraction process developed in this study can be used as basic data for future scale-up and is expected to be one of the ways to sustainably utilize SH. For various industrial applications of SH extracts, biological activities other than antioxidant properties will be evaluated and formulation studies of the extracts will be conducted.

## Figures and Tables

**Table 1:** Design of experiments and their response values (tannin and phenol extraction yield).

Std #	Parameter Symbol (Coded Level)				Responses (mg/g Biomass)	
	A	B	C	D	Tannin Yield	Phenol Yield
1	−1	−1	−1	−1	9.15	7.15
2	1	−1	−1	−1	8.10	5.84
3	−1	1	−1	−1	11.41	9.09
4	1	1	−1	−1	10.74	13.16
5	−1	−1	1	−1	8.54	6.58
6	1	−1	1	−1	7.88	4.50
7	−1	1	1	−1	9.72	4.10
8	1	1	1	−1	8.62	5.29
9	−1	−1	−1	1	10.09	7.54
10	1	−1	−1	1	8.75	11.12
11	−1	1	−1	1	11.49	9.31
12	1	1	−1	1	9.48	12.71
13	−1	−1	1	1	9.18	7.10
14	1	−1	1	1	7.26	5.23
15	−1	1	1	1	9.55	3.43
16	1	1	1	1	8.92	7.69
17	−2	0	0	0	8.95	7.09
18	2	0	0	0	5.59	4.61
19	0	−2	0	0	9.14	6.74
20	0	2	0	0	12.63	9.80
21	0	0	−2	0	12.20	9.02
22	0	0	2	0	8.84	6.81
23	0	0	0	−2	12.17	7.38
24	0	0	0	2	11.63	8.40
25	0	0	0	0	11.24	8.65
26	0	0	0	0	11.73	8.77
27	0	0	0	0	10.14	7.75
28	0	0	0	0	9.92	7.56
29	0	0	0	0	11.58	7.97
30	0	0	0	0	12.04	8.61

Symbols: A, solvent concentration (wt%); B, extraction temperature (°C); C, solid loading (g/L); D, amplitude (%). The extraction conditions are represented as coded levels (see Section 3.2).

**Table 2:** ANOVA results for the initial model for predicting tannin yield from *Sargassum horneri*.

Source	Sum of Squares	DF	Mean Square	F-Value	*p*-Value
Model	71.98	14	5.14	7.9	0.0001
A	10.79	1	10.79	16.59	0.001
B	13.43	1	13.43	20.64	0.0004
C	11.04	1	11.04	16.97	0.0009
D	0.0118	1	0.0118	0.0181	0.8949
AB	0.019	1	0.019	0.0291	0.8668
AC	0.0356	1	0.0356	0.0548	0.8181
AD	0.3673	1	0.3673	0.5646	0.464
BC	0.5944	1	0.5944	0.9136	0.3543
BD	0.4459	1	0.4459	0.6853	0.4207
CD	0.0044	1	0.0044	0.0067	0.9357
A^2^	33.17	1	33.17	50.98	<0.0001
B^2^	1.04	1	1.04	1.59	0.2262
C^2^	2.25	1	2.25	3.47	0.0824
D^2^	0.0958	1	0.0958	0.1473	0.7065
Residual	9.76	15	0.6506		
Lack of Fit	5.91	10	0.5911	0.7682	0.663
Pure Error	3.85	5	0.7695		
Cor Total	81.74	29			

DF, degree of freedom; R^2^: 0.8806, adjusted R^2^: 0.7692, adequate precision: 11.4701. Symbols: A, solvent concentration (wt%); B, extraction temperature (°C); C, solid loading (g/L); D, amplitude (%).

**Table 3:** ANOVA results of the initial model for predicting phenol yield from *Sargassum horneri*.

Source	Sum of Squares	DF	Mean Square	F-Value	*p*-Value
Model	120.18	14	8.58	4.16	0.0048
A	1.64	1	1.64	0.7929	0.3873
B	10.44	1	10.44	5.06	0.0399
C	55.3	1	55.3	26.8	0.0001
D	4.54	1	4.54	2.2	0.1587
AB	13.35	1	13.35	6.47	0.0225
AC	4.24	1	4.24	2.06	0.1722
AD	3.53	1	3.53	1.71	0.2103
BC	15.09	1	15.09	7.31	0.0163
BD	1.84	1	1.84	0.8929	0.3597
CD	0.3811	1	0.3811	0.1847	0.6735
A^2^	9.53	1	9.53	4.62	0.0483
B^2^	0.0061	1	0.0061	0.003	0.9574
C^2^	0.1526	1	0.1526	0.074	0.7894
D^2^	0.1779	1	0.1779	0.0862	0.7731
Residual	30.96	15	2.06		
Lack of Fit	29.6	10	2.96	10.93	0.0083
Pure Error	1.35	5	0.2709		
Cor Total	151.14	29			

DF, degree of freedom; R^2^: 0.7952, adjusted R^2^: 0.6040, adequate precision: 8.8818. Symbols: A, solvent concentration (wt%); B, extraction temperature (°C); C, solid loading (g/L); D, amplitude (%).

**Table 4:** ANOVA results of the reduced model for predicting tannin yield from *Sargassum horneri*.

Source	Sum of Squares	DF	Mean Square	F-Value	*p*-Value
Model	67.19	4	16.80	28.85	<0.0001
A	10.79	1	10.79	18.54	0.0002
B	13.43	1	13.43	23.07	<0.0001
C	11.04	1	11.04	18.97	0.0002
A^2^	31.92	1	31.92	54.83	<0.0001
Residual	14.56	25	0.5822		
Lack of Fit	10.71	20	0.5354	0.6958	0.7458
Pure Error	3.85	5	0.7695		
Cor Total	81.74	29			

DF, degree of freedom; R^2^: 0.8219, adjusted R^2^: 0.7934, adequate precision: 22.6275. Symbols: A, solvent concentration (wt%); B, extraction temperature (°C); C, solid loading (g/L); D, amplitude (%).

**Table 5:** ANOVA results of the reduced model for predicting phenol yield from *Sargassum horneri*.

Source	Sum of Squares	DF	Mean Square	F-Value	*p*-Value
Model	95.83	5	19.17	8.32	0.0001
A	1.64	1	1.64	0.7101	0.4077
B	10.44	1	10.44	4.53	0.0437
C	55.30	1	55.30	24.00	<0.0001
AB	13.35	1	13.35	5.80	0.0241
BC	15.09	1	15.09	6.55	0.0172
Residual	55.31	24	2.30		
Lack of Fit	53.95	19	2.84	10.48	0.0081
Pure Error	1.35	5	0.2709		
Cor Total	151.14	29			

DF, degree of freedom; R^2^: 0.6341, adjusted R^2^: 0.5578, adequate precision: 10.8407. Symbols: A, solvent concentration (wt%); B, extraction temperature (°C); C, solid loading (g/L); D, amplitude (%).

**Table 6:** Optimization results for the efficient extraction of tannin and phenol from *Sargassum horneri*.

A	B	C	D	Tannin Yield (mg/g Biomass)		Phenol Yield (mg/g Biomass)	
				Predicted	Experimental	Predicted	Experimental
39.1	61.9	52.0	49.0	12.95	14.59 ± 1.76	13.37	13.83 ± 0.09

**Table 7:** Antioxidant activity of the *Sargassum horneri* extracts produced under the optimal extraction conditions.

Sample	ABTS IC_50_ (μg/mL)
*Sargassum horneri* extracts	79.86 ± 0.42
Ascorbic acid (reference antioxidant)	54.37 ± 2.18

**Table 8:** Parameters and their levels in the central composite rotatable design of the response surface methodology.

Parameter	Unit	Symbol	Coded Level				
			−2	−1	0	1	2
Solvent conc.	wt%	A	0	20	40	60	80
Extraction temp.	°C	B	30	40	50	60	70
Solid loading	g/L	C	50	75	100	125	150
Amplitude	%	D	20	40	60	80	100

## Data Availability

The original contributions presented in this study are included in the article; further inquiries can be directed to the corresponding authors.

## References

[1] Lee J., Shin H., Lee K.H., Lee H., Lee G., Jang S., Jung G.Y., Yoo H.Y., Park C. (2024). Component analysis and utilization strategy of brown macroalgae as promising feedstock for sugar platform-based marine biorefinery. Biotechnol. Bioprocess. Eng..

[2] Aminina N.M., Karaulova E.P., Vishnevskaya T.I., Yakush E.V., Kim Y.-K., Nam K.-H., Son K.-T. (2020). Characteristics of Polyphenolic Content in Brown Algae of the Pacific Coast of Russia. Molecules.

[3] Indriyawati N., Supriyanto S., Nabwiyah N.D.N. (2024). Nutritional Composition of *Sargassum* sp. and *Padina* sp.. BIO Web Conf..

[4] Han J., Jo Y., Sun H., Lee E., Chae U., Han S.O., Kim J.H., Hyeon J.E. (2023). The Enzymatic Process of Macroalgae for Conversion into High-tech Bioproducts. Biotechnol. Bioprocess. Eng..

[5] El-Sheekh M., Kassem W.M., Alwaleed E.A., Saber H. (2024). Optimization and characterization of brown seaweed alginate for antioxidant, anticancer, antimicrobial, and antiviral properties. Int. J. Biol. Macromol..

[6] Ibrahim R.Y.M., Hammad H.B.I., Gaafar A.A., Saber A.A. (2022). The possible role of the seaweed *Sargassum vulgare* as a promising functional food ingredient minimizing aspartame-associated toxicity in rats. Int. J. Environ. Health Res..

[7] Dolorosa M.T., Nurjanah, Purwaningsih S., Anwar E. (2019). Utilization of *Kappaphycus alvarezii* and *Sargassum plagyophyllum* from Banten as cosmetic creams. IOP Conf. Ser. Earth Environ. Sci..

[8] Shin H.J., Woo S., Jung G.Y., Park J.M. (2023). Indole-3-acetic Acid Production from Alginate by *Vibrio* sp. dhg: Physiology and Characteristics. Biotechnol. Bioprocess. Eng..

[9] Rhein-Knudsen N., Reyes-Weiss D., Horn S.J. (2023). Extraction of high purity fucoidans from brown seaweeds using cellulases and alginate lyases. Int. J. Biol. Macromol..

[10] Shin J., Choi J.G., Kim S.H., Khim B.K., Jo Y.H. (2022). Environmental variables affecting *Sargassum* distribution in the East China Sea and the Yellow Sea. Front. Mar. Sci..

[11] Jeong D.-Y., Jeong D.-I., Jeong S.-M., Kim Y.-J. (2017). Production of Bioethanol via *Sargassum horneri* Fermentation. J. Korean Soc. Urban. Environ..

[12] Kang S.M., Lee C., Jeong D.H., Kim J., Boo K.H., Kim J.H., Kim C.S. (2020). Evaluation of Biological Activities of *Sargassum horneri* Fermented by Microorganisms. J. Korean Soc. Food Sci. Nutr..

[13] Yamaguchi M. (2024). The Marine Alga *Sargassum horneri* Is a Functional Food with High Bioactivity. Nutraceuticals.

[14] Cha W.Y., Byon C. (2022). Development and characterization of an eco-friendly packaging film using *Gelidium amansii* and *Sargassum horneri*. J. Mar. Biosci. Biotechnol..

[15] Hahn D., Kim M.J., Kwon Y., Kim E., Park D.H., Bae J.-S. (2024). Natural products ameliorating the adverse health effects by air particulate matter. Biotechnol. Bioprocess. Eng..

[16] Chen C.J., Han L.S., Yao M.K., Shi L., Zhang M.S., Shi Y.P., Liu C.H., Bai X.F., Liu X., Liu X. (2021). Comparative Studies on Antioxidant, Angiotensin-Converting Enzyme Inhibitory and Anticoagulant Activities of the Methanol Extracts from Two Brown Algae (*Sargassum horneri* and *Sargassum thunbergii*). Russ. J. Mar. Biol..

[17] Kim M.J., Jo H.G., Ramakrishna C., Lee S.-J., Lee D.-S., Cheong S.H. (2021). Anti-inflammatory and antioxidant activities of *Sargassum horneri* extract in RAW264.7 macrophages. Phys. Act. Nutr..

[18] Yamaguchi M., Matsumoto T. (2015). Marine algae *Sargassum horneri* bioactive factor suppresses proliferation and stimulates apoptotic cell death in human breast cancer MDA-MB-231 cells in vitro. Integr. Mol. Med..

[19] Fauzi A., SatrianiLamma M.R. (2018). Total tannin levels analysis of brown algae (*Sargassum* sp. and *Padina* sp.) to prevent blood loss in surgery. Dentomaxillofacial Radiol..

[20] Nie J., Chen D., Lu Y. (2020). Deep eutectic solvents based ultrasonic extraction of polysaccharides from edible brown seaweed *Sargassum horneri*. J. Mar. Sci. Eng..

[21] Han D.F., Zou R.J., Gong X.H., Li J.W., Liu X.J., Luo J.J., Xu Y.J. (2019). Optimization of extraction process for fucoxanthin from Sargassum horneri by response surface methodology. J. Food Saf. Qual..

[22] Yao K.A., Tiho T., Silué N., Assidjo N.E., Koné K.Y. (2023). Total polyphenols, total flavonoids, condensed tannins, and antioxidant activity of *Borassus aethiopum* (arecaceae) ripe fruits’ peels, and peel-pulps, dried at different temperatures. Asian J. Chem. Sci..

[23] Palma-Wong M., Ascacio-Valdés J.A., Ramírez-Guzmán N., Aguirre-Joya J.A., Flores-Loyola E., Ramírez-Moreno A., Torres-León C. (2023). Exploration of Phenolic Content and Antioxidant Potential from Plants Used in Traditional Medicine in Viesca, Mexico. Horticulturae.

[24] Gori A., Boucherle B., Rey A., Rome M., Fuzzati N., Peuchmaur M. (2021). Development of an innovative maceration technique to optimize extraction and phase partition of natural products. Fitoterapia.

[25] Naviglio D., Scarano P., Ciaravolo M., Gallo M. (2019). Rapid Solid-Liquid Dynamic Extraction (RSLDE): A Powerful and Greener Alternative to the Latest Solid-Liquid Extraction Techniques. Foods.

[26] Shin H., Lee J., Bae J., Lee K.H., Yoo H.Y., Park C. (2023). Enhancement of dieckol extraction yield from *Ecklonia cava* through optimization of major variables in generally recognized as safe solvent-based process. Front. Mar. Sci..

[27] Tiwari B.K. (2015). Ultrasound: A clean, green extraction technology. TrAC Trends Anal. Chem..

[28] Hiranpradith V., Therdthai N., Soontrunnarudrungsri A., Rungsuriyawiboon O. (2025). Optimisation of Ultrasound-Assisted Extraction of Total Phenolics and Flavonoids Content from Centella asiatica. Foods.

[29] Shi H., Ling X., Luo X., Su T., Xie X., Ji H., Qin Z. (2024). Mass Transfer Kinetics of Ultrasound-Assisted Steam Distillation for the Extraction of Cinnamon Oils. Korean J. Chem. Eng..

[30] Belwal T., Huang H., Li L., Duan Z., Zhang X., Aalim H., Luo Z. (2019). Optimization model for ultrasonic-assisted and scale-up extraction of anthocyanins from *Pyrus communis* ‘Starkrimson’ fruit peel. Food Chem..

[31] Rodríguez Ó., Bona S., Stäbler A., Rodríguez-Turienzo L. (2022). Ultrasound-Assisted Extraction of Polyphenols from Olive Pomace: Scale Up from Laboratory to Pilot Scenario. Processes.

[32] Gonçalves M.A., dos Santos H.C.L., da Silva M.A.R., da Cas Viegas A., da Rocha Filho G.N., da Conceição L.R.V. (2024). Biodiesel production from waste cooking oil using an innovative magnetic solid acid catalyst based on Ni–Fe ferrite: RSM-BBD optimization approach. J. Ind. Eng. Chem..

[33] Gam D.H., Hong J.W., Jeon S.J., Baek D.H., Kim J.W. (2020). Development of ultrasound-assisted extraction for production of bioactive compounds with whitening and anti-wrinkle effects from *Sargassum horneri*. Korean Soc. Biotechnol. Bioeng..

[34] Kong M.R., Seo S.J., Han M.R., Lee Y.S. (2016). Anti-oxidation and anti-aging activity of three composite species at different extraction temperatures. J. Investig. Cosmetol..

[35] Zhang Y., Prawang P., Li C., Meng X., Zhao Y., Wang H., Zhang S. (2018). Ultrasonic assisted extraction of artemisinin from *Artemisia Annua* L. using monoether-based solvents. Green. Chem..

[36] Dang T.T., Bowyer M.C., Van Altena I.A., Scarlett C.J. (2018). Comparison of chemical profile and antioxidant properties of the brown algae. Int. J. Food Sci. Tech..

[37] Sobuj M.K.A., Islam M.A., Haque M.A., Islam M.M., Alam M.J., Rafiquzzaman S.M. (2021). Evaluation of bioactive chemical composition, phenolic, and antioxidant profiling of different crude extracts of *Sargassum coriifolium* and *Hypnea pannosa* seaweeds. J. Food Meas. Charact..

[38] Shin J. (2024). Fractionation and Structural Characterization of Antioxidant Compounds from Sargassum horneri Ethanol Extract. Master’s Thesis.

[39] Phang S.J., Teh H.X., Looi M.L., Arumugam B., Fauzi M.B., Kuppusamy U.R. (2023). Phlorotannins from brown algae: A review on their antioxidant mechanisms and applications in oxidative stress-mediated diseases. J. Appl. Phycol..

[40] Park S.G., Yang H.K., Yang S.M. (2023). Health Functional Food Composition Containing Extracts of Sargassum Horneri and Exhibiting Anti-Oxidation and Anti-Obesity Effects (1020230042813).

[41] Choung E.S., Lee J.S., Kim J.Y., Han B.N., Jo Y.I., Hong J.H., Lee M.J., Kim D.H. (2018). A Composition Comprising Supercritical Fluid Extract of Sargassum Horneri Having Antioxidant and Skin Whitening Effect (1020180149621).

[42] Li P.-T., Lee Y.-C., Elangovan N., Chu S.-T. (2007). Mouse 24p3 Protein Has an Effect on L929 Cell Viability. Int. J. Biol. Sci..

[43] Turan C.U., Derviscemaloglu M., Guvenilir Y. (2024). Herbal active ingredient-loaded poly(ω-pentadecalactone-*co*-δ-valerolactone)/gelatin nanofibrous membranes. Eur. J. Pharm. Biopharm..

[44] Hemmat Esfe M., Motallebi S.M., Esfandeh S., Toghraie D. (2024). Study of Rheological Behavior, Economic Performance and Development of a Model for MWCNT-ZnO (30: 70)/10W40 Hybrid Nanofluid Using Response Surface Methodology. Korean J. Chem. Eng..

[45] Abubakar A., Manogaran M., Yasid N.A., Othman A.R., Shukor M.Y.A. (2023). Equilibrium, kinetic and thermodynamic studies of the adsorption of trypan blue dye by *Pseudomonas* sp. strain MM02 inactivated biomass. Korean J. Chem. Eng..

[46] Tran T.K., Ha P.T.T., Henry R.J., Nguyen D.N.T., Tuyen P.T., Liem N.T. (2024). Polyphenol Contents, Gas Chromatography-Mass Spectrometry (GC–MS) and Antibacterial Activity of Methanol Extract and Fractions of *Sonneratia caseolaris* Fruits from Ben Tre Province in Vietnam. J. Microbiol. Biotechnol..

